# Academic dishonesty in higher education: students’ perceptions and involvement in an African institution

**DOI:** 10.1186/s13104-016-2044-0

**Published:** 2016-04-25

**Authors:** Sixtus Bieranye Bayaa Martin Saana, Ephraim Ablordeppey, Napoleon Jackson Mensah, Thomas K. Karikari

**Affiliations:** Department of Dispensing Technology, School of Applied Science and Technology, Wa Polytechnic, P. O. Box 553, Wa, Ghana; Department of Building Technology and Estate Management, School of Applied Science and Technology, Wa Polytechnic, Wa, Ghana; Department of Science Laboratory Technology, School of Applied Science and Technology, Wa Polytechnic, Wa, Ghana

**Keywords:** Academic dishonesty, Plagiarism, Cheating, Higher education, Undergraduate, Africa, Ghana

## Abstract

**Background:**

Integrity in academic work is a critical benchmark of every profession. For this reason, special attention should be devoted to addressing academic dishonesty (AD) in higher education to prevent the potential transfer of these practices to the workplace. In order to effectively address AD in Africa, further information about correlates of, and barriers to, the effectiveness of existing AD-controlling measures is needed. In Ghana, little is known about AD from the perspective of students. Here, we present a first report of Ghanaian undergraduate students’ self-reported understanding of, and support for, institutional AD regulations, their involvement in specific dishonest behaviours, as well as their motivation factors.

**Results:**

Approximately 92 % of respondents said they were aware of institutional regulations on AD. However, only 31 % rated their understanding as high. Respondents believed that their lecturers had better understanding of, and support for, these regulations than the students (p < 0.001 and p < 0.0001 respectively). Approximately 40 % of respondents had witnessed their colleagues engage in AD before, but the majority (94 %) had never reported these acts. The pursuit of good grades, high academic load and pressure to please family and guardians were the leading causes of AD. Cheating during examinations and inappropriately sharing answers in the preparation of assignments were some of the highly-occurring forms of AD. Respondents believed that copying colleagues’ work without their permission was a serious offense but doing so with their permission was not.

**Conclusion:**

Our findings suggest that the sampled students consent to cheating—they believed that they committed no misconduct once the parties involved had agreed on the act. Considering these misconceptions, institutions should do more to help their students better understand the different forms of AD and how to avoid them.

## Background

Academic dishonesty (AD) among students can be defined as academic behaviour that does not comply with stated assessment requirements and other institutional policies; when students behave in ways intended to gain undue benefit in relation to their assessment [1]. AD is a global phenomenon occurring in both developed and developing countries [2–7]. Research conducted in different parts of the world has shown that between 40 and 80 % of students in higher education (HE) have been involved in AD at least once [5–10]. In some of these studies, respondents said they had engaged in AD and/or had witnessed their colleagues do so. This is exemplified by the findings of a survey that revealed that 8–39 % of nursing students had been involved in unethical academic behaviours, while the majority (61–94 %) said they had witnessed their peers cheat in the academic environment [8]. A study conducted in Australia and New Zealand found that as high as 342 cases of AD among students had been recorded in fourteen HE institutions within an academic year [3]. In that same study, 6 % of student respondents confirmed being caught engaging in AD. Aside from developed countries, cases of AD have been reported in the developing world. For instance, a study conducted in two Nigerian institutions showed that 54.2 % of undergraduate pharmacy students had been involved in AD in the preparation of their academic exercises [2].

AD tends to be prevalent in institutions with high student to teacher ratios, a situation that is common to many institutions in the developing world [9]. A leading reason for students’ involvement in AD is the pressure to obtain good grades in order to enhance one’s job prospects [8, 10]. AD has also been linked to students’ age and gender; older and female students are significantly less likely to cheat compared to their younger and male counterparts [11, 12]. Students’ lack of awareness of institutional regulations on AD has been reported to also contribute to the problem [13]. Furthermore, factors such as one’s standard of written English, access to scientific literature (both in hardcopy and electronic formats), and the availability of institutional resources and support are potential determinants of students’ inclination to engage in plagiarism. Students with poor command over the English language and those with limited access to reading materials may be inclined to copy the text used in reference materials. In addition, students without anti-plagiarism training and support may engage in unintentional forms of plagiarism. To this end, investigations into AD in low income countries should consider these often-forgotten factors to help differentiate intentional dishonest behaviour from inadvertent unethical behaviour.

AD is a serious problem affecting educational institutions, and therefore needs urgent attention. The need for further studies, particularly in HE institutions, is motivated by the fact that HE is the ultimate level of education from where students are likely to directly enter the job market. Students’ perceptions of what is institutionally acceptable and unacceptable regarding dishonest practices might therefore contribute to their behaviour at the workplace [4]. In fact, students’ inadequate understanding of what constitutes AD has been shown to correlate with the occurence of unethical behaviours [5, 13]. Hence, it is important to identify possible gaps between students’ awareness and understanding of AD and what is stated in their institutional regulations. Unfortunately, the dearth of research data on AD, especially in sub-Saharan Africa, makes it difficult for HE leaders, policymakers and teaching staff to determine the effectiveness of available measures, and what might be needed to improve the existing plans. For example, in Ghana, no published work has explored the possible link between students’ awareness and understanding of institutional policies and the likelihood to engage in academic misconduct. Here, we provide an initial report on students’ self-reported awareness and understanding of institutional regulations on AD, their support for these regulations, their involvement in specific dishonest behaviours, as well as what motivates them to engage in these behaviours. We believe that findings from this study will provide evidence that will help fill the gap between availability and accessibility of AD regulations in HE institutions and the effective implementation of such regulations.

## Methods

We assessed the perceptions of AD and the level of participation by undergraduate students studying Science, Technology, Engineering and Mathematics (STEM) subjects in a HE institution in Ghana. Our focus on STEM students was due to recent efforts by African governments (particularly the Ghanaian government) to improve investments in STEM education to help accelerate the continent’s economic and industrial development [14, 15]. Honesty among the future STEM experts would be a key enabling factor for Africa’s quest for improved development.

### Study site

The study was conducted at Wa Polytechnic (WP), a public HE institution established to train skilled workforce to support Ghana’s economic and industrial growth. WP is located in Wa, the capital of the Upper West region. Established in 1999 by a Presidential Charter, WP is the youngest of the ten polytechnics in Ghana. The institution is administratively divided into four faculties, which in total run ten accredited programmes. The choice of WP for this study was informed by recent high rates of student dismissals due to AD.

This study was conducted among students of the School of Applied Science and Technology, since these students were a good fit for our intention to understand AD among students studying STEM subjects. Students were sampled from three Higher National Diploma (HND) programmes, namely Building Technology & Estate Management, Dispensing Technology (Pharmacy Technology), and Information and Communication Technology (n = 131). HNDs in Ghana are undergraduate programmes but on a slightly lower level compared to similar programmes leading to the award of Bachelor’s degrees (freshmen must have completed and passed 12 years of schooling). The HND concept, modelled after the British system, is aimed at training workforce directly for industries.

### Study procedure and ethical consent

The study involved the design and administration of a structured questionnaire which sought information on students’ age, gender, duration of HE study, and perceptions and possible participation in AD. The questionnaire was designed by faculty members with many years of experience teaching in the institution, and as a result, had had many interactions with students on the topic.

Initially, written approval was obtained from the WP chief examiner. This consent was considered appropriate, having used the proper channels of institutional authority and communication. Respondents were first briefed on the study aims and were encouraged to be as honest as possible in answering questions. Respondents were also assured of confidentiality that information provided would be used solely for the intended study, and would not be used to implicate them. Respondents were at liberty to either opt in or out of the process at any time. Voluntary completion of the questionnaire was deemed to constitute consent from respondents. No personally-identifiable information was recorded. Questionnaires were administered to students in an auditorium, under the supervision of the researchers. Survey data have been used solely for the intended educational research, and processed according to provisions in the Ghana Data Protection Act, 2012 (Act 843; [16]). Since the study was an evaluation of educational experience involving normal classroom practices, we believed that we were not required to seek ethics committee approval.

The questionnaire was divided into two parts i.e., A and B. Part A sought information on students’ awareness and understanding of institutional regulations on AD, and sources of this information. We also obtained data on how students perceived their instructors to understand these regulations. Other information sought included students’ perceptions on the effectiveness and robustness of existing regulations. Also, students’ perceptions on the frequency of occurrence of specific AD practices at the institution, as well as perceived factors that would influence them to engage in AD were sought. In part B, different practices that are often considered as dishonest were provided and students were asked to indicate whether they had previously engaged in any of those practices, assigning a level of seriousness for each practices. Next, we asked students to indicate possible factors that might motivate them to engage in AD. They were also asked to indicate, from a pre-provided list of options, the four most important factors to their education. The aim was to analyse for a potential connection between students’ participation in AD as a means of achieving their educational goals. Demographic data about students were also taken.

### Data analysis

Data were processed using Microsoft Excel 2011 and exported into SPSS 22.0 (IBM Inc., Chicago, USA) and Prism 6 (GraphPad Inc., San Diego, CA, USA) for analysis. Tests of normality, using the Shapiro–Wilk test and the D’Agostino omnibus normality test, showed that the data were non-parametrically distributed. Non-parametric tests were therefore used throughout the analysis. The Mann–Whiteny U-test was used to compare the mean values of two sets of data while binomial logistic regression analysis was conducted to understand if students’ characteristics such as gender, programme and year of study, and reported awareness of AD regulations could be used to predict their understanding of AD measures. Significance was considered achieved at the 95 % confidence interval. Data were reported as mean ± standard deviation.

## Results

### Descriptive statistics about the study respondents

Out of the 131 students sampled, 106 (80.92 %) were males while 24 (18.32 %) were females. One respondent did not indicate gender. Most participants were within the 20–29 years age range (114 students, representing approximately 87 %). Concerning programme of study, the highest percentage of students were enrolled on the HND Building Technology & Estate Management programme (54.96 %), followed by Dispensing Technology (26.72 %) and Information & Communication Technology (17.56 %). There were 68 students (representing 51.91 %) in their 1st year of study, while 45 (34.35 %) and 18 (13.74 %) were in the 2nd and 3rd years respectively. Detailed demographic information about respondents are presented in Table 1.

**Table 1:** Descriptive statistics of study participants

	Sample size	Percentage
Gender		
Male	106	80.92
Female	24	18.32
Not indicated	1	0.76
Total	131	100
Age (years)		
≤19	3	2.29
20–29	114	87.02
30–39	10	7.69
40–49	2	1.53
Not indicated	2	1.53
Programme of study		
Building technology and estate management	72	54.96
Dispensing technology (Pharmacy technology)	35	26.72
Information and communication technology	23	17.56
Not indicated	1	0.76
Year of undergraduate study		
1	68	51.91
2	45	34.35
3	18	13.74

### Students’ awareness of institutional regulations on AD

Students were asked if they had been made aware of institutional policies regarding dishonest academic behaviours, such as regulations on quizzes and examinations. Answers to this question were gathered on a three-point Likert scale, where 1 yes, 2 no, and 3 not sure. Most students (92.37 %) responded that they were aware of such policies; the mean self-reported awareness was 1.09 ± 0.34 (Table 2).

**Table 2:** Students’ self-reported awareness of institutional policies on academic dishonesty

Answer	Number of responses	Percentage (%)	Mean (SD)^a^
Yes	121	92.37 %	1.09 (0.34)
No	8	6.11 %	
Not sure	2	1.53 %	

^a^Responses were taken on a three-point Likert scale; 1 yes, 2 no, and 3 not sure

**Fig. 1:**
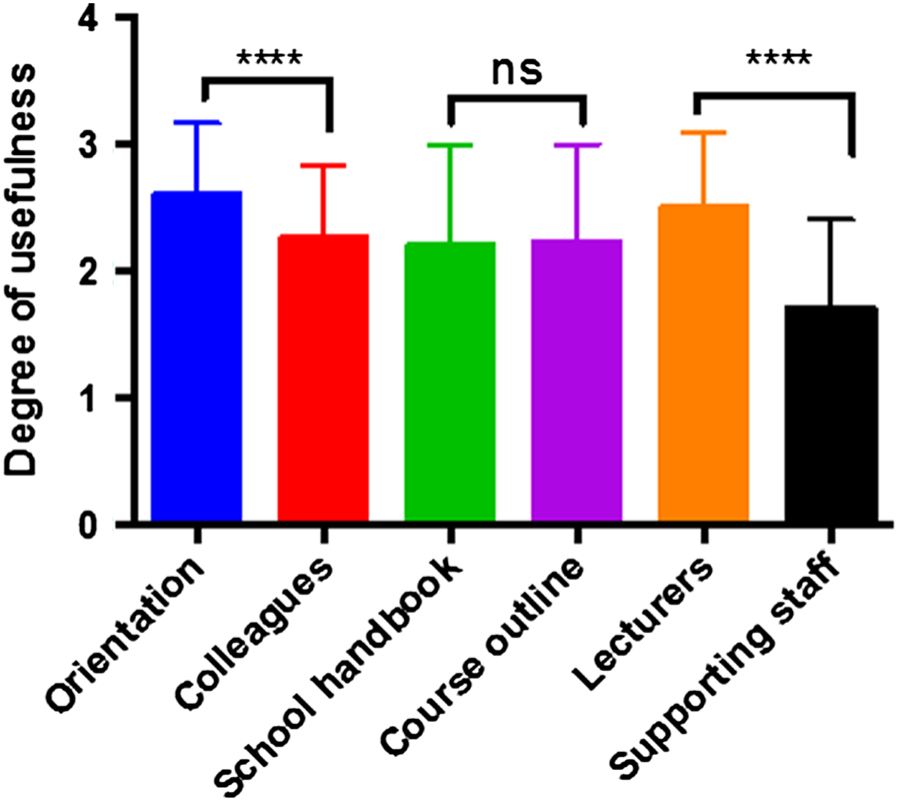
Students’ sources of information on academic dishonesty regulations. Unpaired two-tailed Mann–Whitney U-test, ****p < 0.0001, *ns* not significant at 95 % confidence interval. Respondents found freshman orientation programmes as the most useful source of information on academic dishonesty, followed closely by their lecturers, colleague students, course outlines and students’ handbook. Supporting staff (such as laboratory technicians and teaching assistants were the least useful source of information on dishonest practices). While lecturers were significantly more useful than supporting staff, students’ handbook and course outline were useful to similar extents. Furthermore, freshman orientation programmes were significantly more useful than colleague students regarding information on dishonest behaviours and how to avoid them. Further details can be found in Table 3

Next, we wanted to assess the sources of information on AD guidelines, as well as the extent to which they had found these sources useful. Responses were taken on a three-point Likert scale, with multiple choices allowed (Table 3). Freshman orientation programmes constituted the most useful source of AD-related information, followed by lecturers, friends and classmates, and course outline (Table 3). Supporting staff (e.g. laboratory technicians) were the least useful source (Table 3). Statistical comparison of the usefulness ratings using unpaired, two-tailed Mann–Whitney U-test showed that first-year orientation was a more useful source of AD information than friends/colleagues (p < 0.0001; Fig. 1). Similarly, lecturers were a better source compared to supporting staff (p < 0.0001; Fig. 1). School handbook and course outline were useful to a similar degree (p = 0.7845; Fig. 1).

**Table 3:** Students’ sources of information on institutional regulations on academically unethical behaviour, as well as the usefulness of these sources

Source	Usefulness rating (number of respondents)^a^			Mean rating (SD)
	Not useful	Averagely useful	Highly useful	
Freshman students’ orientation	5	41	79	2.59 ± 0.57
Friends and classmates (colleagues)	8	72	38	2.25 ± 0.57
Students’ handbook	28	39	51	2.19 ± 0.80
Course outline	24	43	52	2.23 ± 0.77
Lecturers	6	48	65	2.50 ± 0.60
Supporting staff	49	52	13	1.70 ± 0.70

^a^Responses were taken on a three-point Likert scale, where 1 not useful, 2 averagely useful, and 3 highly useful

### How well do students understand these policies?

Subsequently, we sought to assess students’ understanding of institutional policies on AD. Students were asked to indicate their levels of understanding of these policies, as well as their perceived effectiveness of the policies and severity of punishment meted out to offenders. Students were also asked to indicate how they perceived their lecturers to understand the AD regulations, and how they believed these lecturers would rank the policy effectiveness and punishment severity. Students and their instructors are major concerned parties when it comes to AD [18]. AD regulations are usually prepared by instructors, with little or no involvement of students. What students think of their instructors concerning these regulations might affect the effectiveness of the regulations. Our survey design allowed us to compare students’ self-reported understanding of AD regulations with the perceived understanding of their instructors. The same four-point Likert scale was used for evaluating students’ (and lecturers’ perceived) understanding and perceived effectiveness of policies and severity of punishment for offenders. This enabled statistical comparison of the different ratings using the Mann–Whitney U-test.

**Fig. 2:**
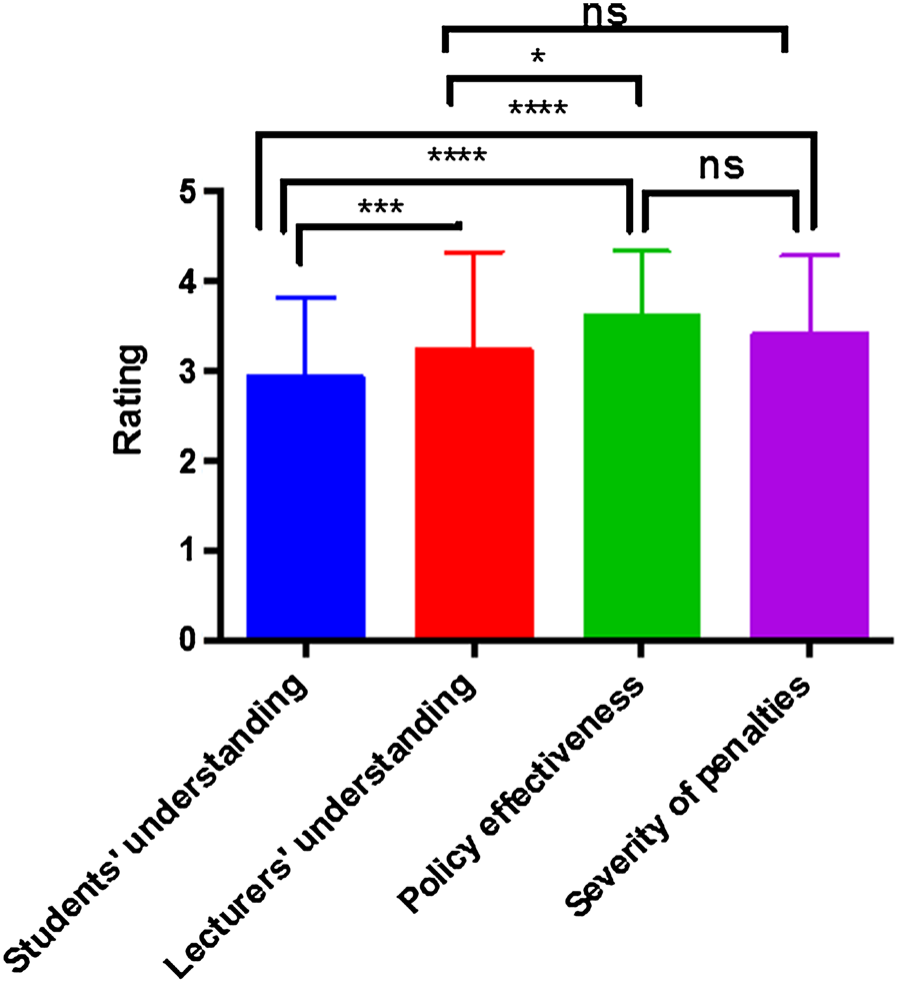
Students’ self-reported understanding and perceived effectiveness of institutional regulations on academically dishonest behaviour, compared with lecturers’ understanding (as perceived by students). Unpaired two-tailed Mann–Whitney U-test *p < 0.05, ***p < 0.001,****p < 0.0001, *ns* not significant at p < 0.05. Students believed that their lecturers significantly better understanding of the regulations. No significant difference was found between the mean perceived effectiveness of the regulations and the mean severity of penalties for offending students. Students rated the perceived effectiveness of the regulations significantly higher than both students’ understanding and the perceived understanding by their lecturers

Students believed that their instructors had better understanding of regulations (mean ratings of 2.92 ± 0.89 and 3.22 ± 1.10 for students and lecturers respectively, p = 0.0008; Table 4; Fig. 2). Only 30.53 % of respondents believed that students had *high* understanding of regulations. On the contrary, 58.78 % of student respondents were of the opinion that lecturers had *high* understanding of the regulations (Table 4). Most students responded that the existing regulations were effective in controlling unethical behaviours (mean rating of 3.59 ± 0.73; percentage of high rating 70.99 %; Table 4). The mean rating for severity of penalties was 3.41 ± 0.87. Mean perceived policy effectiveness was statistically higher than both students’ understanding and lecturers’ perceived understanding of the policies (p < 0.0001 and p = 0.0104 respectively; Fig. 2). While no significant difference was recorded between lecturers’ perceived understanding and severity of penalties (p = 0.3400), the mean rating for penalty severity was significantly higher than students’ understanding of the regulations (p < 0.0001; Fig. 2). No significant difference was recorded between the mean perceived policy effectiveness and severity of penalties (p = 0.0794; Fig. 2).

**Table 4:** Students’ self-reported understanding and perceived effectiveness of institutional AD regulations, compared to how they perceived their lecturers to understand and support these regulations

Question	Mean rating (SD)^a^	Percentage of high ratings^b^
Students’ self-reported understanding of policies	2.92 (0.89)	30.53
Lecturers’ perceived understanding of policies	3.22 (1.10)	58.78
Students’ perceived effectiveness of policies	3.59 (0.73)	70.99
Students’ perceived severity of penalties	3.41 (0.87)	61.83
Students’ support for policies	2.67 (0.83)	18.32
Lecturers’ perceived support for policies	3.23 (1.04)	56.49

^a^Ratings were taken on a four-point Likert scale, where 1 do not know, 2 low, 3 average, 4 high

^b^Percentage of high ratings: number of respondents answering “high” as a percentage of the total number of respondents

### Are students in support of institutional regulations on unethical academic behaviour?

**Fig. 3:**
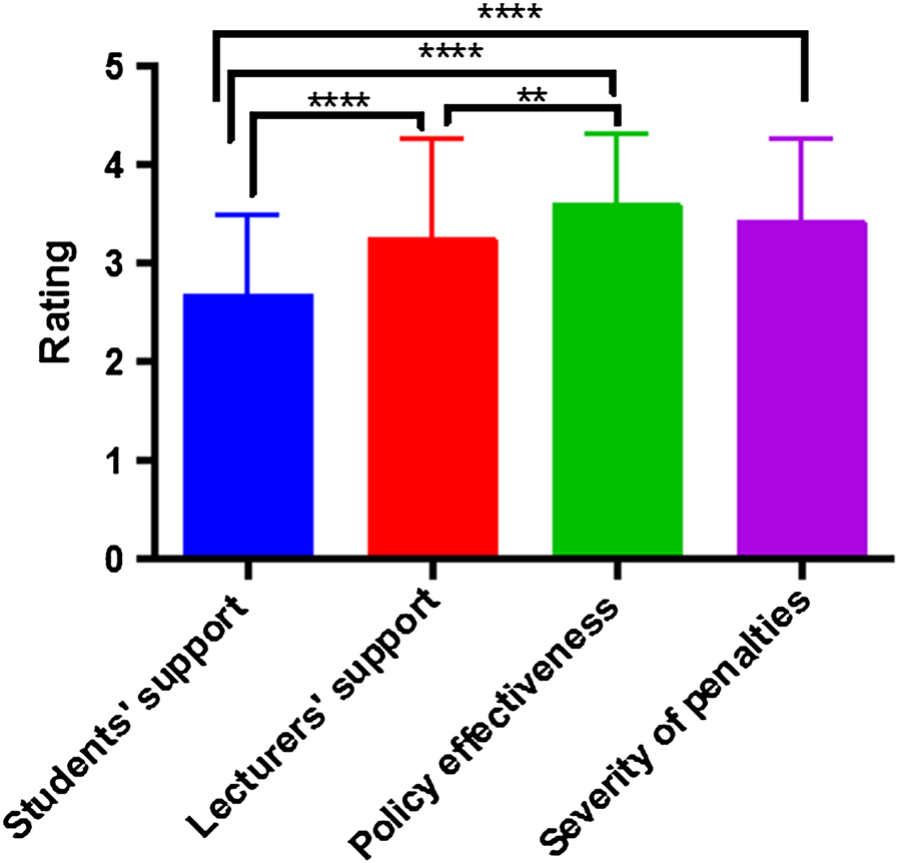
Students’ self-reported support for institutional regulations on academically dishonest behaviour, compared with the perceived support of their lecturers. Unpaired two-tailed Mann–Whitney U-test **p < 0.01,****p < 0.0001. Students believed that their lecturers had significantly better support for the regulations than themselves

For regulations aimed at curbing academic dishonest behaviour to be successful, support from students is crucial. We therefore sought to evaluate the extent to which our study cohort supported their institutional AD regulations, compared to how they perceived their lecturers to support the same regulations. Students’ support was significantly lower than the perceived support from their lecturers (p < 0.0001, mean ratings of 2.67 ± 0.83 and 3.23 ± 1.04 for students and lecturers respectively; Table 4; Fig. 3).

### Occurrence of academically dishonest behaviour, and students’ willingness to report offenders

Students are likely to witness their colleagues engage in unethical behaviours (such as cheating in the examination hall or copying colleague’s assignments) [19]. They may witness these behaviours more often than lecturers will observe their students do same. Therefore, students’ willingness to report offending colleagues would make enormous contributions to ridding the academic environment of unethical behaviours [19]. Respondents were therefore asked to indicate how often they had seen their colleagues engage in specific dishonest behaviours, and the subsequent actions taken (whether they had ever reported cheating colleagues or not).

Most students (approximately 60 %) said they had never witnessed their colleagues cheat in the past (Table 5). Majority (94 %) also responded that they had never reported their colleagues for cheating (Table 5). Regarding perceived occurence of specific unethical academic behaviours, cheating during class tests and examinations, and inappropriately sharing answers in assignments had mostly occurred a few times (mean ratings of 1.93 ± 0.69, 2.01 ± 0.71, and 2.09 ± 0.75; Table 6). Students said that the exchange of money for academic favours was uncommon (mean rating of 1.17 ± 0.39).

**Table 5:** Students’ previous encounter of academically dishonest behaviour among their colleagues and subsequent actions taken

Question^a^	Answer options	No. of respondents (%)	Mean rating (SD)
1. How often have you seen another student cheat during class test or examination?	Never	78 (59.54)	1.55 (0.74)
	Once	33 (25.19)	
	More than once	19 (14.50)	
	Prefer not to say	1 (0.79)	
2. Have you ever reported your colleague for cheating?	Yes	6 (4.58)	1.96 (0.23)
	No	123 (93.89)	
	Prefer not to say	2 (1.53)	

^a^Responses were taken on a four- or three-point Likert scale (questions 1 and 2 respectively)

**Table 6:** Students’ perceived occurrence of specific academically dishonest practices in the study institution

Question^a^	No. of respondents (%)				Mean rating (SD)
	Never	A few times	Many times	Prefer not to say	
Cheating during class tests	35 (26.72)	68 (51.91)	26 (19.85)	2 (1.53)	1.93 (0.69)
Cheating during examinations	32 (24.43)	64 (48.85)	33 (25.19)	2 (1.59)	2.01 (0.71)
Inappropriately sharing answers in assignments	30 (22.90)	56 (42.75)	41 (31.30)	4 (3.05)	2.09 (0.75)
Exchange of money for academic favours	107 (81.68)	19 (14.50)	1 (0.76)	4 (3.05)	1.17 (0.39)

^a^Responses were taken on a four-point Likert scale; 1 never, 2 a few times, 3 many times, 4 prefer not to say

### What factors motivate students to engage in dishonest academic behaviours?

The identification of factors that motivate students to get involved in unethical academic behaviours provides a means to develop effective prevention strategies. To identify factors that might influence students’ involvement in AD, they were asked to separately list factors that motivate them to be dishonest, as well as the most important reasons for their education. We aimed to ascertain if they were engaging in AD as a means to achieve their educational goals.

Students responded that two major issues that motivated them to be dishonest were the pursuit of good academic grades and the pressure not to disappoint family/guardian (Table 7). Interestingly, these same points were recorded as the two most important factors in students’ education (Table 8). These findings suggest that external influences from families and guardians might motivate students to engage in unethical behaviours as a means to achieve good academic records.

**Table 7:** Self-reported factors that motivate students to be dishonest

Motivating factor	Number of responses (%)
Good grades	107 (31.01)
High academic work load	82 (23.77)
Pressure not to disappoint family/guardian	74 (21.45)
Difficulty of subject	73 (21.16)
Lecturers sometimes ignore cheating	4 (1.16)
Find nothing wrong with cheating	3 (0.87)
Cheating is common among students	2 (0.58)

**Table 8:** Most important factors in students’ education

Factor	Number of responses (%)
Getting good grades	117 (24.07)
Pleasing parents/guardian	77 (15.84)
Acquiring knowledge to teach to others	77 (15.84)
Enhancement of job prospects	72 (14.81)
Learning more about the world	50 (10.29)
Interest in programme of study	39 (7.61)
Being challenged to do more	37 (7.61)
Meeting other students	17 (3.50)

### Students’ self-reported involvement in academically dishonest practices

After asking students about their perceived frequencies of occurrence of specific AD practices, we went ahead to ask them about their involvement in some common unethical practices. The most frequently occurring practice was allowing colleagues to copy you, followed by copying colleagues with their permission (mean ratings of 2.24 ± 0.89 and 1.98 ± 0.90 respectively; Table 9). Most students recounted that they had copied their colleagues with permission at least once (mean rating = 1.98 ± 0.90). Only a few students said they had copied friends without permission (mean rating = 1.19 ± 0.58). It would appear that most students consent to cheating; they would copy their friends’ work (with permission) and reciprocate this by helping friends to also cheat. We used the Mann–Whitney U-test to investigate if the occurrences of specific unethical practices differed from each other. We found that practices that occured with similar frequencies were either not significantly different from each other or the degree of significance was low. A notable example was that the two most occurring unethical practices (practices 1 and 4; Table 9) varied from each other by a low significant difference (p = 0.0207). However, the highest occuring practice (allowed colleagues to copy you) varied from all other practices to a higher extent (p < 0.0001; Table 9). It is possible that unethical practices whose occurences were not significantly different from each other (or were significant to small extents) may be related; these practices might either co-occur or the presence of one behaviour might lead to the other. Further studies are required to investigate the dynamics of these behaviours.

**Table 9:** Students’ self-reported involvement in specific academically dishonest practices

	Mean occurence (SD)^a^	Mean serious-ness of positive responses (SD)^b^	Significance of correlations between the mean occurrence of specific academically dishonest practices (Unpaired, two-tailed Mann–Whitney U-test)											
Practice^c^			1	2	3	4	5	6	7	8	9	10	11	12
1. Copying colleagues with permission	1.98 (0.90)	1.19 (0.40)		<0.0001****	<0.0001****	0.0207*	<0.0001****	<0.0001****	<0.0001****	<0.0001****	<0.0001****	0.0197*	<0.0001****	<0.0001****
2. Copying colleagues without permission	1.19 (0.58)	2.00 (0.00)			0.0046**	<0.0001****	0.0205*	0.2165	0.0004 ***	0.9976	<0.0001****	<0.0001****	0.7548	0.3632
3. Failing to participate in group assignments	1.46 (0.86)	1.38 (0.50)				<0.0001****	0.5818	0.1171	0.4930	0.0060**	0.0888	0.0034**	0.0027**	0.0559
4. Allowed colleagues to copy you	2.24 (0.89)	1.32 (0.47)					<0.0001****	<0.0001****	<0.0001****	<0.0001****	<0.0001****	<0.0001****	<0.0001****	<0.0001****
5. Helped others to cheat	1.41 (0.86)	1.43 (0.50)						0.2865	0.2038	0.0228*	0.0165*	0.0003***	0.0107*	0.1611
6. Used electronic device to access the Internet during assessment	1.33 (0.79)	1.43 (0.54)							0.0231*	0.2269	0.0007***	<0.000****	0.1339	0.7470
7. Whispered or signalled answers to others during exams	1.66 (1.99)	1.59 (0.50)								0.0006***	0.3143	0.0223*	0.0002***	0.0087**
8. Sent foreign materials to the exam hall	1.24 (0.69)	1.63 (0.52)									<0.0001****	<0.0001****	0.7586	0.3699
9. Disobeyed examination timing instructions (e.g. continuing to write after the alloted time was over)	1.57 (0.81)	1.35 (0.49)										0.1558	<0.0001****	0.0002***
10. Had prior knowledge of examination questions	1.73 (0.90)	1.19 (0.40)											<0.0001****	<0.0001****
11. Asked a lecturer for favour when marking scripts	1.23 (0.69)	1.50 (0.55)												0.2307
12. In any other way cheated	1.29 (0.75)	2.00 (0.00)												

^a^Mean values out of four possible answer choices, where 1 never, 2 once, 3 more than once, 4 not applicable. SD standard deviation

^b^Average seriousness ratings of positive responses (responses of having previously engaged in a specific practice *once* or *more than once*). Seriousness rating was taken on a two-point Likert scale, where 1 not serious, and 2 serious

^c^The numbering of practices (both horizontal and vertical) are identical. (Unpaired two-tailed Mann–Whitney U-test, **** p < 0.0001, *** p < 0.001 and ** p < 0.01, * p < 0.05)

### Students’ perceived seriousness of dishonest behaviour

The seriousness that students attach to potentially unethical behaviours may affect whether they engage in those practices or not. We asked students to indicate their perceived seriousness for each of the potential AD practices listed in Table 9. These responses were filtered to only include responses from students who had been involved in such behaviours at least once, enabling the researchers to understand how such students regarded the consequences of their actions. An interesting finding was that students were of the view that copying their colleagues’ work with permission was not a serious offense but doing so without permission rather constituted a serious offense (mean rating of 1.19 ± 0.40 and 2.00 ± 0.00 respectively). This finding further supports the idea that the students consented to cheating– they believed that once the parties involved had agreed on the act, they did not commit any misconduct.

### Inter-relationships between hypothesised predictive factors and students’ understanding of AD rules

In order to identify potential socio-demographic determinants of students’ understanding of AD regulations, a binary logistic regression was performed. In this analysis, the dependent variable was students’ understanding of AD measures while the independent variables were age, gender, awareness of AD regulations, programme of study, and previous reporting of AD cases (Table 10). To obtain two categories of outcome for the dependent variable, responses showing *no* or *low* understanding of AD rules were pooled into one category, and *medium* to *high* understanding into another category (Tables 4, 10). The model explained 14.4 % (Nagelkerke R^2^) of the variance in students’ understanding of AD regulations and correctly classified 64.9 % of cases. Females were 2.021 times more likely than males to understand AD regulations. Increasing age was associated with a reduced likelihood of understanding AD policies. No significant relationships were identified between students’ age, gender, programme and year of study, awareness and previous reporting of AD cases and their likelihood to understand AD measures.

**Table 10:** Socio-demographic characteristics of students’ understanding of institutional regulation on academically unethical behaviour: a binary logistic regression analysis

Dependent variable: students’ understanding of AD measures					
Independent variable	Total respondents (%)^a^	Understanding of AD regulations [number (%)]^b^		p value	OR (95 % CI)
		No to low [n = 44 (34.92 %)]	Medium to high [n = 82 (65.08 %)]		
Age				0.999	
≤19	3 (2.29 %)	0 (0 %)	3 (100 %)	1.000	1.753 × 10^9^ (0.000−)
20–29	114 (87.02 %)	42 (37.17 %)	71 (62.83 %)	0.999	1.350 × 10^9^ (0.000−)
30–39	10 (7.69 %)	2 (22.22 %)	7 (77.78 %)	0.881	1.250 (0.067–23.164)
40–49	2 (1.53 %)	0 (0 %)	1 (100 %)	0.806	1.487 (0.062–35.434)
Gender					
Male	106 (80.92 %)	34 (32.69 %)	70 (67.31 %)	1.000	1.082 (0.000−)
Female	24 (18.32 %)	10 (50 %)	10 (50 %)	0.179	2.021 (0.724–5. 639)
Awareness of AD regulations				0.979	
Yes	121 (92.37 %)	39 (33.05 %)	79 (66.95 %)	1.000	2.545 × 10^9^ (0.000−)
No	8 (6.11 %)	3 (37.5 %)	5 (62.5 %)	1.000	2.169 × 10^9^ (0.000−)
Programme of study				0.919	
Building technology and estate management	72 (54.96 %)	25 (35.21 %)	46 (64.79 %)	1.000	1.665 × 10^9^ (0.000−)
Dispensing technology (pharmacy technology)	35 (26.72 %)	10 (29.41 %)	24 (70.59 %)	0.540	1.624 (0.344–7.655)
Information and communication technology	23 (17.56 %)	9 (40.91 %)	13 (59.09 %)	0.354	2.217 (0.412–11.933)
Previous reporting of AD cases				0.991	
Yes	6 (4.58 %)	2 (40 %)	4 (60 %)	1.000	751146336 (0.000−)
No	123 (93.89 %)	41 (34.17 %)	79 (65.83 %)	1.000	0.731 (0.000−)

*OR* odds ratio

^a^Percentages do not add up to 100 % since some respondents provided neutral answers (see Tables 1, 2)

^b^Total number of respondents for AD regulation understanding across rows do not always add up to the total number of sampled respondents because some respondents did not answer the question on AD understanding

## Discussion

In this study, we have shown that making students aware of institutional AD regulations does not necessarily mean that they will understand such measures. While most students were aware of AD regulations, only about a third adequately understood these regulations. Students believed that their lecturers had significantly better understanding of these regulations, and supported the regulations significantly more than the students. Although most students had previously witnessed their colleagues cheat, they failed to report such acts. The pursuit of good grades and high academic work load were the leading factors motivating students to engage in AD. These were also the two most important factors to students’ education, suggesting that students would cheat to achieve their academic goals.

While most students were aware of institutional AD regulations, only a few adequately understood these regulations (Tables 1, 2). This might be because students in many African institutions (including the study site in the present report) are often told of AD rules and penalties for violations. However, training programmes to teach students about the different forms of AD (such as failing to properly reference information) and practical means to avoid such behaviours are often lacking [20, 21]. This lack of training programmes might affect students’ understanding of, and involvement in, AD [20, 21]. Although we did not investigate the specific aspects of institutional AD policies that students were aware of or understood, it appears that many students in similar African settings are better aware of regulations concerning conduct in examination centres and penalties for violating such regulations compared to guidelines about information referencing [22]. For example, Theart and Smit found in a survey of South African students that about 20 % more students were aware of regulations concerning assessment venues than information referencing [22]. HE institutions should recognise that students are likely to engage in unethical behaviours if they do not adequately understand why they should not do so [5, 13]. Students with better knowledge of AD regulations cheat less [23]. The focus therefore should be on providing students with practical measures to identify and avoid unethical behaviours, instead of waiting for them to do so and “punish” them [20, 24]. Moreover, the finding that most students perceived their lecturers to have significantly better understanding and support for AD regulations (Table 4; Figs. 2, 3) can be explained by the fact that such regulations are usually prepared by faculty members, with minimal or no involvement of students. It might be fair to assume that students will naturally not support measures that will prevent them from engaging in unaccepted behaviours to get ahead academically. But with improved understanding of why they should not do so, the situation might improve [20, 21, 25]. The identification of freshman orientation programmes as the most useful source of AD-related information (Table 3; Fig. 1) could be because such programmes are often highly patronised by students; institutions can use this platform to support new students with information in academic ethics and honesty [20]. This training should however not end here but should be a continuous provision for all students throughout their period of study. To achieve this, purpose-designed training approaches (such as workshops and seminars) should be developed and offered regularly to support students in the practical aspects of ensuring academic honesty.

In many developed countries, detection and control of AD (such as plagiarism in assignments and teaching students how best to avoid them) has been made easier through the application of plagiarism-detecting software products [26, 27]. In developing countries where such technologies are lacking, the problem is often compounded by high student to teacher ratios that have been shown to promote AD [21, 28]. In the midst of these challenges, acts of AD may go unnoticed, and opportunities to help students avoid future occurrences would be missed [21]. The most highly occurring dishonest practices were cheating during class exercises and examinations, and the inappropriate sharing of academic information (Table 6). We speculate that these behaviours might happen due to the following: (1) high student to lecturer ratios, which means that lecturers often have high numbers of student assignment and examination scripts to mark within a short period of time, making it difficult to detect plagiarism and other forms of AD (2) the lack of plagiarism-detecting software in the study institution, making it almost impossible for instructors to identify plagiarism cases, and (3) the lack of closed circuit television devices in examination halls to complement invigilators’ efforts in preventing cheating [21, 28]. Other studies conducted elsewhere have found dishonesty relating to assignments as a highly occurring form of AD among students [22, 29, 30].

While a large proportion of students had previously seen their mates cheat academically, almost none had reported such acts (Table 5). This might be because students cheat on their friends’ works and return this favour by allowing friends to copy their work (Table 9). Hence, all such students had engaged in wrongdoing, making them feel they did not have the moral right to report others for the same act. On the other hand, it is possible that the students believed in group-oriented cultural practices where seeing friends’ work with permission may be acceptable, as reported by others [31, 32]. In group-oriented societies, helping weaker students, even on individual assignments, is an acceptable act [32]. Therefore, the understanding of AD may differ from one culture to the other. However, it is presently unknown as to whether such cultural factors influence Ghanaian students’ decision to plagiarise. Future research into this may help to ascertain if plagiarism is dependent on specific cultural factors in the Ghanaian society. Our findings on students’ opinions concerning cheating on friends’ with or without permission (Table 9) are similar to those of Brown [8] which reported that most students had previously seen their peers cheat. McCabe (2009) also reported that students regard cheating in assignments as a non-serious form of AD [30]. Institutions should therefore develop approaches aimed at providing practical measures to help students better understand and avoid unethical acts.

Moreover, the tendency to cheat may be explained by the limited access to literature resources. Compared to developed countries, the study cohort only had access to hardcopy library textbooks, with no institutional access to research publications online. This might have predisposed them to copying the text in the limited materials at their disposal. In addition, an often-overlooked point in AD research in Africa is students’ background and the learning environment. In several institutions (including the study institution here), learning by memorising information in a textbook or lecture notes and repeating it verbatim during examination is accepted and even encouraged. Furthermore, the requirement for students to cite their sources of information in essays is sometimes not enforced by instructors. Plagiarism therefore becomes acceptable in such institutions, meaning that students who engage in it may do so inadvertently.

We identified (1) the pursuit of good grades (2) high academic workload, and (3) pressure to please family/guardian as leading motivation factors for cheating (Table 7). Obtaining good grades and pleasing family/guardians were the most important factors in their education (Table 8). We infer that students engage in AD for two major reasons: (1) to obtain good grades in order to enhance their job or further education prospects (2) to please family members that their investments (financial, social, and other forms of investment) in the students’ education have paid off. These findings mean that AD is a complex problem, with far-reaching consequences beyond the academic environment. Multifaceted approaches should therefore be employed in addressing the problem.

In the logistic regression analysis, we found that females were twice more likely than males to understand AD regulations, although no significant relationship was recorded (Table 10). While some previous studies reported non-significant relationships between gender and tendency to cheat [22, 30, 33], many others identified males as more likely to engage in AD [18, 29, 34] with one reporting that females were more inclined to be dishonest [35]. These suggest that gender-dependence of cheating might be environment-specific. Increasing age was associated with a reduced likelihood of understanding AD policies, meaning that younger students were more likely to engage in dishonest acts. We found no significant relationships between students’ age, gender, programme and year of study, awareness and previous reporting of AD cases and their likelihood to understand AD measures. An earlier report found no significant relationship between tendency to engage in AD and year of study [22].

## Limitations

The major limitation of this study is the relatively small sample size (n = 131), making the generalisation of findings difficult. Moreover, the study was conducted among students in a single institution, suggesting that some responses may be institution-specific and may therefore not apply to other institutions in Ghana or other developing countries.

For these reasons, we urge readers to exercise caution in extending our results and conclusions.

## Conclusion

AD is a complex challenge which is difficult to address, particularly in educational settings where technological aids used in detecting and preventing dishonest acts are lacking. The approach described herein provides a practical means of evaluating the effectiveness of institutional measures against dishonest academic practices in Ghana and beyond. This work provides evidence that many Ghanaian students do not adequately understand and follow institutional rules on AD; neither do they fully appreciate the consequences of dishonest actions. Institutions should therefore devise more innovative means of training students to avoid dishonest behaviours. This would ensure that industry and society maintain confidence in academic institutions as being able to raise future leaders with integrity.

## Abbreviations

AD: academic dishonesty
HE: higher education
HND: higher national diploma
STEM: science, technology, engineering and mathematics
WP: Wa Polytechnic
